# The impact of nutrition on the lives of patients with digestive cancers: a position paper

**DOI:** 10.1007/s00520-022-07241-w

**Published:** 2022-06-27

**Authors:** Marianna Vitaloni, Riccardo Caccialanza, Paula Ravasco, Alfredo Carrato, Aleksandra Kapala, Marian de van der Schueren, Dora Constantinides, Eva Backman, David Chuter, Claudia Santangelo, Zorana Maravic

**Affiliations:** 1 Digestive Cancers Europe, Brussels, Belgium; 2 UOC Dietetics and Clinical Nutrition, IRCCS San Matteo Polyclinic Foundation, Pavia, Italy; 3 Medicine and Scientific Research, Catolica Medical School & Centre for Interdisciplinary Research in Health - Universidade Católica Portuguesa (UCP); Centre for Interdiscipinary Research Egas Moniz, (CiiEM), Egas Moniz Cooperativa de Ensino Superior, CRL, Lisbon, Portugal; 4 Pancreatic Cancer Europe, Brussels, Belgium; 5 Alcalá University, Madrid, Spain; 6 Clinic of Oncological Diagnostics, Cardio-Oncology and Palliative Medicine, National Oncology Institute Maria Skłodowska-Curie, State Research Institute, Warsaw, Poland; 7 HAN University of Applied Sciences, Wageningen University & Research, Wageningen, Netherlands; 8 PASYKAF, the Cyprus Association of Cancer Patients and Friends, Nicosia, Cyprus; 9 The PALEMA Cancer Society, Lidingö, Sweden; 10 Vivere Senza Stomaco Si Puó, Ferrara, Italy

**Keywords:** Patient experience, Quality of life, Nutritional intervention, Nutrition, Digestive cancers

## Abstract

Nutritional intervention is an essential part of cancer treatments. Research and clinical evidence in cancer have shown that nutritional support can reduce length of hospitalisation, diminish treatment-related toxicity, and improve nutrient intake, quality of life, and physical function. Nutritional intervention can improve outcomes and help patients in the successful completion of oncological treatments by preventing malnutrition. Malnutrition is a very common hallmark in patients with cancers. Almost one-fourth of cancer patients are at risk of dying because of the consequences of malnutrition, rather than cancer itself. Patients with digestive cancers are at higher risk of suffering malnutrition due to the gastrointestinal impairment caused by their disease. They are at high nutritional risk by definition, yet the majority of them have insufficient or null access to nutritional intervention.Inadequate resources are dedicated to implementing nutritional services in Europe. Universal access to nutritional support for digestive cancer patients is not a reality in many European countries. To change this situation, health systems should invest in qualified staff to reinforce or create nutritional teams’ experts in digestive cancer treatments. We aim to share the patient community’s perspective on the status and the importance of nutritional intervention. This is an advocacy manuscript presenting data on the topic and analysing the current situations and the challenges for nutrition in digestive cancers. It highlights the importance of integrative nutrition in the treatment of digestive cancers and advocates for equitable and universal access to nutritional intervention for all patients.

## Introduction: cancer, nutrition, and malnutrition

Cancer is a multidimensional disease requiring multimodal care able to integrate several therapies and treatments. Nutrition is considered a supportive therapy for cancer patients. A large body of existing evidence shows the benefits of professional nutritional support in improving nutrient intake, quality of life, and physical function in patients with cancer. Nutritional intervention can play a central role in the successful completion of oncological treatments and outcomes, also improving final prognosis by preventing and treating malnutrition associated with the disease and its treatment [1]. Prevention of malnutrition is key during a patient’s cancer journey, yet there is a significant disparity across Europe in terms of access to nutritional intervention for cancer patients.

Currently, an accepted gold standard for the diagnosis of malnutrition in cancer is lacking, with data suggesting up to 87% of patients can develop malnutrition, and 15 to 40% of patients reporting weight loss already at diagnosis [2]. Malnutrition in cancer is associated with fewer opportunities for surgical treatment, prolonged hospitalisation, higher treatment-related toxicity, reduced dose-density in radiation and medical oncology treatments, diminished response to cancer treatment, lower activity levels, impaired quality of life, and a worse overall prognosis [3]. Research suggests that up to 20% of cancer patients may die because of the consequences of malnutrition, rather than cancer itself [4].

Malnutrition can manifest in a range that goes from obesity to underweight. The latter of which can progress to cachexia, a specific form of malnutrition driven by a negative energy balance with an increased basal metabolic rate and inflammation. It is characterised by loss of lean body mass, weight, muscle wasting, impaired immune, physical, and mental function [5]. Both conditions of obesity and cachexia often remain overlooked. Cachexia, even when diagnosed, is untreated in about 50% of cases, and obese patients are not properly evaluated to disregard malnutrition [6–8]. Another serious condition related to malnutrition is sarcopenia, characterised by a decrease in muscle mass with a negative impact on strength and physical function and diminished basal metabolic rate that can decrease quality of life. Accumulating evidence shows it may increase treatment toxicity and consequently contribute to worse prognosis [9]. Sarcopenia is often overlooked in obese or overweight patients, while these persons are experiencing changes in body composition that may increase metabolic risk [8, 10].

Minimal weight loss during chemo/radiotherapy has been associated with reduced survival [11]. During chemo/radiotherapy, over 50% of patients experience adverse side effects that can impair food and nutrient intake [12]. The side effects of cancer and its treatment such as anorexia, early satiety, nausea, vomiting, oral and intestinal mucositis with dysphagia, diarrhoea, and unpleasant modification in smell and taste not only affect total energy consumption but also the absorption of nutrients, compromising optimal nutritional status. Nutritional intervention can reduce adverse side effects and help patients cope with and finish their treatment plan.

By nature, digestive cancers are associated with gastrointestinal tract impairment. Patients with digestive cancers are one of the most vulnerable groups that suffer from malnutrition and its consequences. Digestive Cancers Europe (DiCE) is the European umbrella organisation, representing the voice of patients with digestive cancers (oesophageal, gastric, colon, rectum, liver, pancreatic and rare cancers), their families, and their carers from thirty-two countries.

It is the mission of Digestive Cancers Europe to contribute to early diagnosis and decreased mortality from digestive cancers and to increase overall survival and quality of life. This position paper aims to emphasise the importance of integrative nutrition in the treatment of digestive cancers and advocates for equitable and universal access to nutritional intervention for all patients.

## The relevance of nutritional intervention for cancer patients

The food we consume and the nutrients we get from our daily diet have a profound impact on our health status and wellbeing. Healthy nutritional habits can prevent diseases, delay their onset, and improve symptoms and outcomes. In cancer, research has shown that nutritional support may improve the overall experience of patients during their journey. Nutritional intervention encompasses nutrition counselling and education, oral nutritional supplements, and enteral and/or parenteral nutrition support, and is recognised as a supportive therapy that plays a significant role in cancer treatment [13].

The benefits of nutritional intervention for cancer patients are several and significant. Nutritional intervention can improve weight status, energy, and protein intake, and reduce nutrition negative impact symptoms [13]. Treatment side effects and toxicity can be reduced while survival and global outcome are improved. Preventing malnutrition can reduce hospital length of stay, decrease treatment costs, and increase their performance [6, 14].

Nutritional support and physical exercise are key to preventing muscle mass loss and maintaining functionality and physical activity. Besides the clinical aspects and physiology, nutrition is also an important factor for mental and social health. It helps in modulating anxiety, preserving social connections that are often built around experiences related to food sharing, and educating on health habits. Overall, patients with access to nutritional counselling have an improved quality of life [15, 16].

Patients should have access to free and early nutritional screening as a first step to ensure the benefits that personalised nutrition intervention can achieve for their lives, families, and caregivers. The evaluation of the nutritional status of each patient should be a multimodal process based on personal characteristics and preferences, clinical history, treatments conducted, in progress and planned, uncontrolled symptoms, detection of anthropometric parameters, and laboratory tests.

An organisational model capable of ensuring adequate, timely, effective, efficient, and safe nutritional interventions for patients must be based on interdisciplinary and multi-professional working groups in which the various professionals work in a closely specialised organisation. These types of models have been implemented already in some European health systems, such as The Netherlands and Sweden. Although, in Europe, they represent the exception to the rule [17].

## Nutrition in digestive cancers

People affected by digestive cancers are one of the most vulnerable groups at risk of suffering nutritional complications [18]. The functioning of the digestive tract is often mechanically and physiologically altered by the presence of a malignant tumour(s) or by the surgical intervention. Gastrointestinal dysfunction and inflammation caused by the tumour(s) can impair food intake and nutrient absorption, causing dysphagia, pain, and vomiting, with many patients reporting weight loss at the time of diagnosis.

Because of their pathophysiology, all patients with digestive cancer are at high nutritional risk. Some digestive cancers are associated with a fast and deep detrimental weight loss that can interrupt or complicate their treatment and ultimately deny them access to vital surgery. Patients with digestive cancers should have access to nutritional support from the moment of their diagnosis. Ensuring a timely and dynamic nutrition assessment process, designed by nutritional experts in collaboration with all healthcare professionals involved in their treatment plan, is key to avoiding malnutrition. This process must integrate the patient’s preferences and needs and be adaptable to the various phases of their journey.

Malnutrition in digestive cancers is associated with a poor prognosis. Over two decades ago, Kelsen and colleagues reported weight loss in oesophageal cancers as a predictor of poor outcome and associated it with higher mortality risk [19]. Alves and colleagues confirmed that weight loss of over 10% in patients undergoing colon surgery is an independent preoperative risk factor of mortality [20]. On the contrary, preoperative oral immunonutrition for gastrointestinal cancer patients has been associated with diminished risk of postoperative infectious complications and overall hospital stay [14]. Early diagnosis of malnutrition can significantly reduce the number and length of post-surgical stays in patients with digestive cancer, confirming that early nutritional therapy has the potential to significantly improve outcomes [21].

A clinical trial conducted by Ravasco and colleagues comparing three groups of patients with colorectal cancer showed that individualised nutritional counselling, education, and monitoring, along with timely dietary management of symptoms improved, among other factors, nutritional and non-nutritional outcomes, such as quality of life, disease progression, treatment toxicity, and mortality. The study results highlighted that these positive effects were maintained during long-term follow-up, which suggests that personalised nutritional intervention can have a lasting beneficial effect on the health and wellbeing of patients in the long term [22].

There is an urgent need for expert nutritionists and dieticians to assist in the dynamic treatment of digestive cancers. Although digestive cancers share a range of common characteristics, specific nutritional intervention is required based on the organ affected and the localisation of the tumour in the gastrointestinal tract.

## Nutritional intervention and digestive cancers: current situation and challenges

Nutritional intervention for digestive cancer patients is not a reality in most European countries. Insufficient resources are dedicated to implementing nutritional services that cannot be properly applied. There is a strong need for nutritionists and dietitian experts on gastrointestinal cancer treatments to work in coordination with other healthcare professionals.

Despite the key role of nutrition support in cancer treatment in some countries, such as Poland, nutritionists and dieticians are not even recognised as health professionals. And in countries where they are, the number of professionals dedicated to providing nutritional counselling and designing nutritional intervention is, in most cases, insufficient to cover the user’s needs. Consequently, oncologists and nurses must face the gap by providing nutritional advice and assuming the burden of a task that is not in their full competence and for which they have not received adequate training. There is a clear need to strengthen the role of nutritionists and dietitians and to train physicians and nurses on the basics of nutrition for oncology patients.

Misinformation is of critical concern regarding the importance of nutrition when treating patients with cancer. In many countries, clinicians are still not totally aware of the impact of nutritional intervention on the life and health of patients. Even if most clinicians see malnutrition as a risk factor for increased morbidity and mortality, nutrition management of digestive cancer patients remains lacking [23, 24]. The lack of clear, easily accessed, and evidence-based guidelines specific to digestive cancer patients creates a burden for clinicians to advocate for nutritional support. Besides the complexity of current guidelines, there is a lack of useful practicable criteria, a multiplicity of these criteria, and a lack of publications on large data [25]. Future guidelines should incorporate the patient’s perspective and be designed together with patients.

The strategy of nutritional intervention must include the patient’s perspective, building a critical discussion interchange between the patient and the nutrition expert. Patients are experts of their own conditions; nutritional intervention should incorporate their preferences based on their stories, needs, and concerns. The European societies embrace different cultures and traditions within the same national borders. Nutritional intervention should be personalised considering the strong relationship between diet and social-cultural aspects.

The nutritional approach requires closeness and practicality. Digestive cancer patients in treatment require weekly monitoring, with evidence showing that nutritional counselling and intervention incorporated at the time of diagnosis can reduce treatment toxicity and hospitalisation rates, thereby lowering health systems expenses [22, 26–29]. However, these services are currently inaccessible to all patients due to the lack of financial resources and professional experts.

Access to nutritional counselling allows patients to build a relationship and bond based on trust and familiarity with their nutritionist or dietitian, they become a credible and trusted reference for patients and families, preventing them from following unproved diets and misleading nutritional advice. Patients who lack access to nutritional help within their healthcare system tend to search for private nutritional support, relying, in some cases, on uncertified professionals who may enlist interventions that could endanger their health or disrupt their treatment [30].

The bond of trust, once created, sets the basis for an open dialogue that can help health professionals and patients make crucial decisions together. For example, addressing concerns about artificial nutrition. Artificial nutrition therapies, both parenteral and enteral nutrition, are useful when used to support patients during their oncologic treatments and can improve their quality of life. These therapies are a key tool to ensure nutritional intake and are widely used in gastrointestinal cancers. Artificial nutrition needs to be managed by expert nutritionists working closely with oncologists in the context of dynamic nutrition counselling that begins at diagnosis and where patients’ preferences and needs are at the centre of the discussion.

Access to a nutritional team should be agile and patients need to feel there is an open door to communicate with their nutritionists and dieticians. The feeling of continued nutritional and clinical support can improve a patient’s quality of life and should be a key component throughout their journey. Nutritional counselling is also a moment of education and empowerment for the patient. They learn what their body needs to stay healthy from an expert and this knowledge can serve as a basis for maintaining a healthy lifestyle during treatment and the stages of remission and afterwards.

Educating patients and families on nutrition, and how to maintain a healthy lifestyle, creates the basis for them to know how to take care of their diet throughout their lives. Social and cultural aspects of food should be considered, and the knowledge and perception of healthy eating should be evaluated. The creation of nutrition skills for patients, caregivers, and families will also be important during the survivorship period. At this stage, digestive cancer survivors have less access to nutritional counselling, although they still need support. Structures providing nutritional intervention for cancer survivors are extremely important to ensure a good recovery, quality of life, and social and economic rehabilitation. Still, the gap between supply and demand is huge. Nutritional counselling and education can prevent obesity during the survivorship period, one of the major risk factors for cancer relapse [22, 30, 31].

A recent Irish study surveying more than a thousand cancer survivors confirmed that patients consider nutrition support extremely important. Nearly half of the respondents suffer from diet-related problems. The group of gastrointestinal cancer patients reported a higher incidence of nutritional problems, unintentional weight, and muscle loss. Respondents said they could not visit a dietician because they were not referred, with more than half of them wanting to receive more dietetic support. Over one-third of cancer survivors were following unproven dietary strategies, and a majority felt confused by nutritional advice in the media [30].

The major challenge for most European countries is investing in qualified staff to reinforce their health system’s nutritional teams so that they can ensure free, early, and easy access to nutritional intervention services for patients in treatment for digestive cancers.

With a major barrier being a lack of information and dialogue on the impact of nutrition on cancer among healthcare professionals and policymakers, additional challenges ahead for nutrition in digestive cancer are to:

- Guarantee total reimbursement of oral supplementations for all patients. Even in regions where patients have access to nutritional counselling, they often must pay out of pocket for the supplements they need to live. Sometimes, in gastrotomised patients, life-long supplements are required.
- Provide correct prescription and monitoring of artificial nutrition at home considering patient’s preferences and using standardised-quality criteria at no additional costs for patients and their families.
- Integrate protocols for patients with digestive cancer by reformulating the urgency for these patients to access prehabilitation programs.
- Study the patient’s body composition to better detect malnutrition. A CT scan or a bioimpedance are valid options.
- Design easy access and evidence-based guidelines ensuring the integration of the patient’s perspective.
- Establish new nutritional guidelines and recommendations for immunotherapy treatments and other new drugs based on novel technologies.
- Increase awareness in healthcare professionals and the general population on the beneficial role of nutrition in cancer and for a healthy lifestyle.
- Provide training in nutritional assessment and counselling to all healthcare professionals related to the cancer patient.
- Collect and analyse data and create registries on the impact of nutritional support in cancer to study their cost-effectiveness on a broader scale.
- Increase the research on malnutrition, sarcopenia, cachexia, and cancer where several factors are involved. Many relevant knowledge gaps exist in our understanding of these conditions.

## Conclusions

Nutrition is a central aspect in oncology, influencing the development of the disease, cancer symptoms, response to, and recovery after treatment(s) as well as improving prognosis of the disease. It has a powerful impact on the quality of life of patients, both at the clinical and social levels, modulating anxiety and preserving personal connections.

Access to integrative nutritional support should be guaranteed to all cancer patients and mostly to those who are at higher risk of malnutrition, as for patients with digestive cancers. Nutritional status is not a fixed condition, but a dynamic status. Therefore, patients’ nutritional conditions need to be assessed periodically during the different phases of the treatment journey, ensuring stable and continuous access to nutritional interventions.

Several barriers prevent patients from benefitting from nutritional intervention in Europe. The lack of investment in qualified personnel to join nutritional teams and the under-recognised impact of nutritional support among oncologists and policymakers prevent patients from benefiting from a life-saving therapy.

There is a strong need for educating the medical and the patient communities on the importance of nutrition in digestive cancers. For this, new educational and scientific resources focusing on nutrition in cancer and targeting healthcare professionals and the general public are needed. Nutritional intervention can be implemented to encompass patients’ needs and requests. The creation of an open dialogue between healthcare professionals, policymakers, other stakeholders, and patients to learn about their lived experiences, stories, and perspectives can no longer be postponed. Incorporating patients’ viewpoints in implementing nutritional intervention can improve healthcare services, reduce clinical costs, and most importantly, increase the quality of life of thousands of people and families.

## Data Availability

Not applicable.

## Declarations

### Disclaimer

The funders had no role in study design, data collection and analysis, decision to publish, or preparation of the manuscript.
